# Polycystic Ovary Syndrome: Pathophysiology and Controversies in Diagnosis

**DOI:** 10.3390/diagnostics13091559

**Published:** 2023-04-26

**Authors:** Duaa Fahs, Dima Salloum, Mona Nasrallah, Ghina Ghazeeri

**Affiliations:** 1 Department of Obstetrics and Gynecology, Faculty of Medicine, American University of Beirut Medical Center, Beirut P.O. Box 113-6044, Lebanon; 2 Division of Endocrinology and Metabolism, Faculty of Medicine, American University of Beirut Medical Center, Beirut P.O. Box 113-6044, Lebanon

**Keywords:** polycystic ovary syndrome, diagnostic criteria, pathophysiology

## Abstract

Polycystic ovary syndrome (PCOS) is a complex and heterogeneous disorder that commonly affects women in the reproductive age group. The disorder has features that propose a blend of functional reproductive disorders, such as anovulation and hyperandrogenism, and metabolic disorders, such as hyperglycemia, hypertension, and obesity in women. Until today, the three implemented groups of criteria for the diagnosis of PCOS are from the National Institutes of Health (NIH) in the 1990s, Rotterdam 2003, and the Androgen Excess Polycystic Ovary Syndrome 2009 criteria. Currently, the most widely utilized criteria are the 2003 Rotterdam criteria, which validate the diagnosis of PCOS with the incidence of two out of the three criteria: hyperandrogenism (clinical and/or biochemical), irregular cycles, and polycystic ovary morphology. Currently, the anti-Müllerian hormone in serum is introduced as a substitute for the follicular count and is controversially emerging as an official polycystic ovarian morphology/PCOS marker. In adolescents, the two crucial factors for PCOS diagnosis are hyperandrogenism and irregular cycles. Recently, artificial intelligence, specifically machine learning, is being introduced as a promising diagnostic and predictive tool for PCOS with minimal to zero error that would help in clinical decisions regarding early management and treatment. Throughout this review, we focused on the pathophysiology, clinical features, and diagnostic challenges in females with PCOS.

## 1. Introduction

Polycystic ovarian syndrome (PCOS), described as an ovarian dysfunction, is a heterogeneous reproductive disorder with hormonal and metabolic implications. It is a common endocrine disorder that directly causes anovulatory infertility in females of reproductive age [1]. The prevalence of PCOS ranges between 4% and 20% depending on the set of diagnostic criteria used [2]. Irregular menstrual cycles, weight gain, hypertension, diabetes, and infertility are all PCOS symptoms.

The pathogenesis of PCOS is multifactorial, involving an interplay among genetic, environmental, and trans-generational factors. As a result, the clinical spectrum of PCOS involves interrelated metabolic, reproductive, and psychological impairments [3,4]. The main hormones contributing to the development of PCOS are estrogen, androgen, and the anti-Müllerian hormone (AMH). Due to the heterogeneity of PCOS, its diagnosis and management are challenging as the leading symptoms vary with age and presentation [5]. A 20-year follow-up study of women with PCOS by Carmina et al. and Jolanda et al. identified phenotypic changes associated with PCOS, including an increase in ovulatory cycles and a decrease in serum androgen levels, luteinizing hormone levels, and ovarian volume [6,7].

The vast array of possible diagnostic criteria, treatment routes, and often incompatible recommendations have led to international medical controversy. The three diagnostic tools for PCOS are the (1) the National Institute of Health 1990, (2) Rotterdam 2003, and (3) the Androgen Excess-PCOS Society 2006 criteria. Currently, the Rotterdam criteria are the most commonly used diagnostic tool [3,8]. In 2020, Dokras et al. reported that despite 85% of obstetrics and gynecology residents using the internationally accepted Rotterdam criteria to diagnose PCOS, less than 10% were able to identify the five components of the criteria [9].

The standard diagnostic tool for PCOS is transvaginal ultrasound; however, this is accompanied by preprocessing downsides, such as the speckle noise reduction problem [10]. Unwanted speckle noise in ultrasounds results from the interference of positive and negative spread signals resulting from the human body. Consequently, noise in an ultrasound contributes to reduced image contrast [11]. Based on various research studies and relevant clinical queries, artificial intelligence (AI) is being proven as a promising technology for PCOS diagnosis involving predictive models that aid clinicians in diagnosis and treatment [12,13,14]. AI methods can use innovative machine learning and advanced algorithms to understand features from a large dataset in order to develop a valid diagnostic framework for PCOS [15,16,17].

## 2. Pathogenesis of PCOS

### 2.1. Hyperandrogenism

Hyperandrogenism is a multifactorial PCOS pathology influenced by a combination of environmental and heritable elements. Hyperandrogenism can result from an imbalance in the hypothalamus–pituitary–ovarian axis signaling process, leading to excess secretion of insulin and luteinizing hormone [18]. Another cause can be theca cells’ intrinsic dysfunction or diminished levels of cortisol that stimulate negative feedback on the hypothalamic–pituitary axis and elevate the synthesis of the hypothalamic adrenocorticotropic hormone after adrenal steroidogenesis stimulation, a leading factor in adrenal hyperandrogenism [19,20,21].

Clinically, hyperandrogenism manifests as hirsutism, androgenic acne, and/or alopecia resulting from elevated circulating androgen levels [22,23]. While there is no entirely accepted visual assessment for an androgenic acne diagnosis, the degree and distribution of alopecia can be evaluated using the Ludwig visual score. Hirsutism is diagnosed using the modified Ferriman Gallwey score (MFG), which is the standard for the clinical assessment of hirsutism. The score defines hirsutism as the overgrowth of male-type hair in the nine androgen-sensitive areas of a woman’s body: the upper lip, chin, chest, upper and lower back, upper and lower abdomen, upper arm, and thighs [24]. The score starts from 0 (no terminal hair growth) to 4 (male pattern hair growth) in each of the nine areas and requires a minimum score of 8 to diagnose hirsutism. A diagnostic dilemma regarding using the MFG score for clinical hyperandrogenism diagnosis has arisen, because the score is examiner-dependent and has been shown to have decent intra-observer reliability, but inadequate inter-observer reliability [25].

When the clinical signs, especially hirsutism, are absent or unclear, a biochemical assessment of hyperandrogenism is vital for the diagnosis of PCOS. Hyperandrogenism is characterized by increased levels of testosterone of ovarian origin, high levels of androstenedione, or increased levels of adrenal androgens dehydroepiandrosterone and dehydroepiandrosterone-sulfate [26]. Notably, another diagnostic dilemma arises here because laboratory assays are initially tailored and calibrated to measure androgen levels in males. However, calibration studies have not been conducted to develop a female androgen assay. In addition, biochemical evaluation is unreliable in the case of women on hormonal contraception due to impacts on sex hormone-binding globulin and distorted gonadotrophin-dependent androgen production [27].

### 2.2. Insulin Resistance and Hyperglycemia

Insulin is primarily responsible for glucose homeostasis and lipogenesis. Women with PCOS have intrinsic insulin resistance at a prevalence of 12–60% regardless of the degree of obesity or the level of androgens [28]. Insulin resistance is defined as a pathological condition characterized by decreased responsiveness or sensitivity to the metabolic actions of insulin [29,30]. Insulin resistance plays an influential role in the development and persistence of PCOS. This is mainly due to a defect in insulin receptors resulting from excessive serine phosphorylation and decreased tyrosine phosphorylation, which leads to a decrease in insulin activation of the phosphatidylinositol-3-kinase signaling pathway that activates glucose transport and consequently increases glucose levels [31,32]. Increased insulin secretion directly triggers the pituitary gland to release luteinizing hormone, which triggers androgen secretion and affects the development and growth of ovarian follicles [33,34]. Both elevated insulin and androgen levels inhibit sex hormone-binding globulin (SHBG) secretion, which leads to an increase in free and bioactive androgens [35].

Insulin resistance measurement is complicated; thus, several tests have been developed to measure this condition. While more readily usable and simple tests are less precise, some other tests are more complex, but also more reliable. The gold standard for direct measurement of metabolic insulin sensitivity is the hyperinsulinemic euglycemic glucose clamp method [36]. Specifically, it quantifies the amount of glucose metabolized by the body in response to a hyperglycemic stimulus. The method is based on a presumption that after a continuous insulin infusion, hepatic glucose production will be completely suppressed by the generated hyperinsulinemic state, while there will be no net change in the steady-state glucose level [37]. However, this method is not frequently used because of its complexity as a procedure.

### 2.3. Anti-Mullerian Hormone

Anti-Müllerian hormone (AMH) is a glycoprotein that belongs to the transforming growth factor-β (TGFβ) superfamily [38,39]. The production of AMH begins 36 weeks after conception and its level peaks in neonatal life, after which it remains low until reaching the puberty phase. In adolescence, AMH levels rise to reach a plateau, followed by a dramatic drop before menopause. After menopause, the hormone becomes undetectable. The highest expression of AMH is recorded in preantral and small antral follicles 2–4 mm in diameter; however, it can also be expressed by growing follicles up to 8 mm [38,40]. AMH inhibits primary follicle recruitment and may be involved in protecting growing follicles from premature maturation [41].

Women diagnosed with PCOS have higher AMH levels than normal women, which leads to the possibility of using AMH as a surrogate marker for the diagnosis of PCOS [42,43,44]. The elevation is induced by an increase in the number of preantral follicles and small antral follicles, thus leading to increased secretion within each of these follicles [45]. However, the increase in AMH levels is not only due to the increased number of preantral and small antral follicles, because elevated AMH levels have been detected in both anovulatory PCOS cases and normal-ovulatory PCOS cases in comparison to normal non-PCOS cases. While no explanations for AMH overproduction have been identified to date, a positive correlation has been found between androgens and AMH expression [46,47]. In women with PCOS, the overexpression of AMH and AMH type II receptors on granulosa cells may also be responsible for the overexpression of AMH [48,49].

Although AMH has been related to ovarian follicle count and is considered an ovarian reserve marker, serum AMH levels have not yet been introduced as an alternative tool for the detection of polycystic ovary morphology (PCOM), nor as a single test for the diagnosis of PCOS. AMH’s accuracy has shown significant heterogeneity in studies, making it unsuitable for clinical use. There is emerging evidence that with the improved standardization of assays and established cut-off levels or thresholds based on large-scale validation in populations of different ages, AMH assays will be more accurate in the detection of PCOM. Precisely, (1) defined general population-based samples instead of choosing subjects from high-risk populations, along with (2) well-defined cut-off values for AMH and (3) a clearer definition of PCOM, are three crucial steps that would benefit the cause [50].

## 3. Other Clinical Features of PCOS

### 3.1. Metabolic Syndrome

The modified American Heart Association/National Heart Lung and Blood Institute AHA/NHLBI (ATP III 2005) defined metabolic syndrome as having three or more of the following metabolic symptoms: (1) obesity (waist circumference of ≥88 cm), (2) hypertension (blood pressure ≥130/85 mm Hg), (3) dyslipidemia (high-density lipoprotein (HDL) of ≤50 mg/dL), and (4) hyperglycemia (fasting blood sugar of ≥100 mg/dL). The prevalence of metabolic syndrome among women with PCOS was 45.8%, as found by Madhusudaran et al. [51]; 46%, as reported by Glueck et al. [52]; and 43.4%, as reported by Ishak A et al. [53]. It was more prevalent in hyperandrogenic PCOS phenotypes in comparison to normal androgenic phenotypes.

The metabolic symptoms of PCOS seem to be connected; androgen excess is the beginning of a vicious cycle of metabolic disorders in PCOS patients. A positive correlation was observed between PCOS diagnosis in women and overweight and abdominal fat deposition [54]. A study in 2017 by Melal et al. included 1387 women previously diagnosed with PCOS and reported at least 52% of them to be struggling with obesity [55]. Hyperglycemia is yet another metabolic disorder associated with PCOS. Findings from longitudinal cohort studies have reported that developing hyperglycemia is significantly more common in PCOS-diagnosed women within the age group of 15 to 49 years old compared to non-PCOS control women [56,57]. Dyslipidemia, reflected by high triglycerides and low HDL cholesterol, is one of the most common metabolic disorders identified in females with PCOS [58,59]. In multiple cross-sectional studies, PCOS has been linked to significantly higher blood pressure compared to normal controls, independently of weight/obesity [60,61].

### 3.2. Reproductive–Infertility

It is noteworthy that women with PCOS are prone to hormonal imbalances and ovulatory disturbances, leading to infertility. In PCOS, fertility is adversely affected by anovulation, increased risk of spontaneous abortion, poor quality of oocytes, elevated serum LH concentration, and hyperinsulinemia-linked miscarriages [62,63,64].

Women with PCOS have poor reproductive and pregnancy outcomes and are at a higher risk of endometrial hyperplasia related to ovulatory dysfunction, as well as infertility. While PCOS relates to lower pregnancy rates, this is not related to the number of parities in those women. Multiple studies conducted worldwide have shown that PCOS is the most conventional trigger of female factor infertility [27,65]. In a cross-sectional study conducted by Joham et al., infertility was recorded in 72% of women with PCOS compared to 16% in women without PCOS. In addition, the study discovered significantly higher use of hormonal fertility treatments among women with PCOS [66]. Moreover, Bahri et al. presented evidence that women with PCOS have at least a twofold risk factor for miscarriage, pregnancy-induced hypertension, hyperglycemia, and pre-eclampsia compared to control women without PCOS [67].

As a result of disrupted ovulatory function, women with PCOS are recommended to follow oral ovulatory induction therapies, such as letrozole [68]. Even though they are less effective, lifestyle adjustments alongside the recommended therapies may boost ovulation frequency and propose a potential adjunct. Upon pregnancy, PCOS patients are also at a higher risk of gestational diabetes and preeclampsia when compared to control females without PCOS [27,69].

## 4. Challenges in the Diagnosis of PCOS and Different Criteria

### 4.1. NIH Criteria

The National Institutes of Health (NIH) international conference was the first conference on PCOS that anticipated diagnostic criteria for PCOS in the early 1990s [70]. These were based on a combination of two criteria: (1) oligo-anovulation and (2) clinical or biochemical signs of hyperandrogenism. Both criteria must be present after excluding all other androgen excess-related or anovulatory infertility-related diseases. Further analytical studies revealed additional characteristics of PCOS that were then evaluated by the European Society of Human Reproduction and Embryology (ESHRE) and the American Society for Reproductive Medicine (ASRM), and the Rotterdam criteria were proposed in 2003.

### 4.2. Rotterdam Criteria

The Rotterdam consensus includes three diagnostic criteria and requires the presence of two out of the three to confirm PCOS diagnosis. The Rotterdam criteria include (1) oligo-ovulation or anovulation, (2) clinical/biochemical signs of hyperandrogenism, or (3) polycystic ovary morphology (PCOM). Based on the Rotterdam criteria, PCOS cases are distinguished into four different phenotypes based on the presence and/or absence of the three diagnostic criteria (Table 1).

Unlike the other criteria, the Rotterdam criteria do not require the presence of irregular menstrual cycles as a crucial symptom for PCOS diagnosis, but rather considers women with hyper-androgenesis and PCOM as PCOS cases [71,72]. The rationale behind this diagnostic consensus is to widen the inclusion criteria and to recognize that PCOS does not represent a particular entity, but rather occurs in a range of heterogeneous disorders, in addition to the fact that associated long-term health risks, such as type 2 diabetes mellitus and cardiovascular diseases, are commonly encountered in women diagnosed with PCOS [73,74].

While the Rotterdam criteria distinguish between PCOS patients based on their anovulatory pattern, hyperandrogenemia, and PCOM (two out of three), they fail to take into consideration the metabolic status of the patients, which is sometimes reflected in increased body mass index (BMI) and obesity in some women with PCOS [75].

### 4.3. Androgen Excess–PCOS (AE-PCOS) Society 2006 Criteria

The most recent diagnostic criteria of PCOS were compiled by the Androgen Excess and PCOS Society (AE-PCOS) in 2009, which deeply re-examined diagnostic features of PCOS, including menstrual irregularities, hyperandrogenism, and PCOM. A modified version with a balance between the NIH criteria and the Rotterdam criteria was introduced, including three criteria: (1) hyperandrogenism; (2) ovarian dysfunction, including oligo-anovulation and/or PCOM; and (3) exclusion of other androgen excess-related disorders [76]. Specifically, disorders to exclude are Cushing’s disease, 21-hydroxylase-deficient congenital adrenal hyperplasia, thyroid disorders, premature ovarian failure, and androgen-secreting neoplasms.

The AE-PCOS criteria are similar to the NIH criteria in that they consider androgen excess as a necessary component for PCOS diagnosis. Unlike the Rotterdam criteria, PCOM with ovulatory dysfunction (Phenotype D) alone does not qualify a patient for diagnosis according to the AE-PCOS criteria (Table 2). Hence, the AE-PCOS criteria are more inclusive than the NIH version, but less so than the Rotterdam criteria.

## 5. Limitations of the Currently Used Diagnostic Criteria

The Rotterdam criteria are most widely used for PCOS diagnosis; however, they do not take into consideration the difference between adult and adolescent female physiology. Particularly, diagnosis in adolescents can be challenging due to the overlap of diagnostic features of PCOS with normal puberty physiology. As such, the Rotterdam criteria would result in an over-diagnosis of adolescents with PCOS [77], suggesting that further research should be conducted and modifications should be made to the diagnostic criteria in adolescents [27,78]. For adolescents with PCOS, two essential criteria are irregular menstrual cycles and clinical and/or biochemical hyperandrogenism [27]. The guideline also recommends that PCOM should not be considered a criterion for PCOS diagnosis during the first 8 years of menarche. If only one of the two criteria is met, adolescents should be treated as cases at high risk of PCOS and should receive adequate medical follow-up and symptom management [79].

While the Rotterdam criteria grant the diagnosis of PCOS based on PCOM and chronic anovulation without evidence of hyperandrogenism, the NIH and AE-PCOS criteria perceive hyperandrogenism as the center of the PCOS diagnosis process [80]. The heterogeneity in the prevalence estimates for each set of criteria reflects not only the potential differences between study populations, but also the broad clinical spectrum of the condition and the lack of standardization of the cutoffs for each set of diagnostic criteria. In their 2017 study, Ding et al. reported that the heterogeneity between diagnostic criteria is a source of over and/or underdiagnosis of PCOS [81]. Due to the limitations, further research is required to identify a more objective test.

Despite advances in ultrasound technology, the identification of PCOM remains challenging with the variation in the standards used to report the follicle count cutoffs, given that the current threshold of 12 or more follicles is sufficient to diagnose PCOM. As technology has improved, it has become possible to see/identify more follicles, so the previous cutoffs are no longer valid. A systematic review that followed the international evidence-based guidelines, including 11 studies with 2961 participants, analyzed ultrasounds for the follicular number per ovary criterion for PCOM identification, and concluded that the optimal follicular number per ovary to be used was ≥20 follicles per ovary in at least one of the ovaries [27]. In addition, the AE-PCOS society published guidelines in 2014 recommending that the threshold of follicular number per ovary be set at ≥25 when using up-to-date ultrasound technologies that offer a maximal resolution of ovarian follicles [82].

In the case of teenage patients, ultrasonographic evaluation of ovarian morphology might not be possible. In this setting, transvaginal ultrasound might be inaccessible due to virginity combined with possible insufficient imaging by abdominal ultrasound due to abdominal obesity. Moreover, the multi-cystic appearance of ovaries in teenagers is yet another limitation in diagnosing PCOM [83].

Multiple factors, such as diet, stress management, BMI, and the perceived stress of the illness, lead to the exacerbation of PCOS symptoms. Modifications to the patient’s lifestyle are necessary to deal with the side effects of the disease, including the mental burden [84,85]. Although PCOS has relatively well-defined clinical, biochemical, and ultrasound-based markers in adult females, the symptoms in adolescents may overlap with those of normal puberty, making a diagnosis challenging. It is crucial to differentiate between conventional adolescence and real ovarian hyperandrogenism, both of which are correlated with the risk of other health conditions such as diabetes type 2 and cardiovascular disorders [37,86].

## 6. Artificial Intelligence in PCOS Diagnosis

By definition, AI is a blend of reasoning, language understanding, and problem-solving perception, among other features. It is currently being utilized in the healthcare field to obtain better results and decisions by decreasing human error. Beneficially, researchers are applying AI to automatically classify ultrasound images, including transvaginal ultrasounds [87,88]. Through collecting and analyzing clinical data, AI is capable of learning and extracting characteristics to diagnose PCOS, with the ability to provide results with minimal to no error and to disregard unwanted data.

Nowadays, AI is branching into various subsets. Among them is machine learning, which is predominantly spreading in the healthcare field. Machine learning-based AI provides not only an early detection tool, but also a promising predictive model for clinical application [89]. Designing promising and accurate machine learning models is based on keystones of computational and data algorithms for computing data, learning underlying patterns, and drawing useful knowledge for decision making [89,90,91]. In particular, machine learning is used in PCOS to identify its stage and the state of both the uterus and fallopian tube by ultrasound techniques. It also aids in detecting antral follicular count and follicular size [12,16].

AI techniques, including various machine learning models, have shown hopeful markers for the precise and accurate clinical diagnosis of PCOS [12,16]. Machine learning effectively introduces a well-defined diagnostic mechanism with minimal human error and high efficiency in providing optimum patient care [88,92]. Bharati et al. presented a data-driven study on PCOS diagnosis by applying machine learning algorithms [12]. Conducted on 541 women, the study suggested that follicle-stimulating hormone (FSH) and LH were important markers, detected accurately and with lower computation times compared to 43 other markers [12]. Another study by Silva et al. investigated 58 different variables and suggested lipid accumulation product, abdominal circumference, and FSH to be among the important variables associated with PCOS using BorutaShap and random forest algorithms [93].

For instance, the random forest algorithm is a machine learning algorithm proposed by Breiman as an effective tool for regression and classification. This tool classifies datasets randomly into two categories: the first category is “training data” for learning, and the second category is validation data for “testing the learning level” in the random forest algorithm. Following that, decision trees are created whereby randomly picked predictors at node locations define the branching of each tree. The final estimate of the random forest algorithm is the average of all of the tree’s results. As a result, for certain weights, each individual tree has an impact on the random forest estimation. Due to its ability to arbitrarily accept training data from subsets and to set up trees with arbitrary methods, the random forest algorithm surpasses other machine learning algorithms [12]. Furthermore, since training is performed on different randomly selected sub-datasets using bootstrap sampling, the random forest algorithm maintains a level of overfitting [94,95].

Highly efficient machine learning algorithms such as the Random Forest algorithm automatically classify PCOS patients into categories according to their clinical patterns [93]. This classification would help in future investigations related to PCOS pathogenesis and, thus, would improve personalized treatment approaches [12].

To ensure its high value in practice, extensive studies are being conducted on AI-aided software that employ deep machine learning-based segmentation models of ovary/follicle imaging [96]. The software, which can be loaded into the ultrasound equipment, aids in recognizing small-sized follicles and overcoming the preprocessing noise. In particular, one approach could be based on two major functional phases: the preprocessing phase and the follicle identification phase. Follicles appear in low-echo areas inside the contours of the ovaries, which make them difficult to identify. To address this, the preprocessing phase selects a region of interest and identifies all the possible candidates using machine learning-based classifiers such as object-growing algorithms and boundary vector fields [97]. Combining the results of these classifiers with patient diagnostic information could yield a promising PCOS diagnostic tool for use in clinic [98]. Therefore, this imaging tool has the potential to become a valuable diagnostic service for PCOS patients.

## 7. Future Remarks

Leptin is a hormone originating from fat cells that plays a crucial role in regulating glucose homeostasis. Obese patients have elevated leptin levels, leading to diminished sensitivity to leptin receptors and increased leptin resistance. Elevations in leptin may impact follicular development and fertility, and are linked to PCOS [99,100]. Excessive leptin causes triglyceride accumulation in adipose tissue, the pancreas, and the liver, triggering impaired insulin sensitivity, which leads to insulin resistance [101]. In a study investigating the association between leptin and PCOS, Peng et al. demonstrated a significant association between PCOS and leptin levels [99]. In particular, patients with PCOS had higher leptin levels compared to the controls, and an association between leptin levels and PCOS-related hyperandrogenism and insulin resistance was concluded. However, the interaction between leptin, androgen, and insulin in the pathogenesis of PCOS is still vague; thus, leptin’s role as a predictive marker of PCOS has yet to be established [102,103,104,105].

Adiponectin is an adipocytokine expressed by adipose tissues. Similar to leptin, adiponectin has an effect on metabolic disorders such as insulin resistance, type 2 diabetes, and obesity [106]. It plays a role in the reproductive system by inhibiting both LH and gonadotropin-releasing hormone (GnRH) secretion. Consequently, adiponectin is crucial for modulating the central reproductive axis. A study by Boshku et al. demonstrated the presence of low adiponectin levels in a group of women with PCOS compared to a healthy control group [107]. The study also indicated a positive correlation between adiponectin and LH, as well as adiponectin and LH/FSH ratio, due to adiponectin being tangled when gonadotropin release is disturbed in women with PCOS [107]. Another study by Onyegbule et al. highlighted that adiponectin levels were also observed to be much lower in obese/overweight women with PCOS in comparison to in normal-weight women with PCOS [108]. The study refers to this finding as an inverse relation between increased adiposity/BMI in obese patients and reduced adiponectin secretion [109]. This suggests the possible involvement of serum adiponectin in the pathogenesis of PCOS and introduces adiponectin as another possible PCOS biomarker in correlation with obesity [110,111].

In a recent genetic investigation, multiple loci were found close to genes participating in ovarian function, gonadotropin production, and metabolism [88]. Despite the phenotypic variation among PCOS-diagnosed females, extensive research on one genetic location, the DENND1A gene, led to its role in ovarian steroids’ origin. Even though AMH has long been thought to significantly contribute to ovarian dysfunction, recent research studies on animals have shown that AMH stimulates LH release and increased gonadotropin-releasing hormone. These findings suggest a link between AMH and endocrine instability [112,113].

Multiple genes have been correlated with PCOS throughout the years due to its multifactorial etiology. Because a variety of proteins and signaling pathways are involved in the pathogenesis of PCOS, a single genetic diagnostic approach has not yet been explained. The aforementioned entities, such as hyperandrogenemia, hyperinsulinemia, insulin resistance, anovulation, abnormal ovarian morphology, and metabolic disorders, may have a hereditary or epigenetic origin. Existing studies are being conducted to identify genetic markers that could be used in the future for the diagnosis of PCOS [105,114,115].

## 8. Conclusions

The pathophysiology of PCOS is heterogeneous and complex in nature. Despite its prevalence in reproductive-aged women, the identification of PCOS remains challenging due to its uncertain pathogenesis. Currently, PCOS is prevalent not only in women of reproductive age, but also among adolescent girls. It is a significant risk factor for metabolic disorders and is thus linked to the development of type 2 diabetes. Women with PCOS face hyperandrogenism and insulin resistance, among other complications, leading to reproductive and metabolic abnormalities. In addition, anomalous AMH is emerging as a factor in PCOS pathophysiology; however, it has not yet been incorporated as an adequate diagnostic test. Herein, we discuss some of the challenges, controversies, and limitations of the current diagnostic tools. We shed light on future markers that would allow for more timely and accurate diagnoses, and which will mitigate the complications that lead to infertility. Using artificial intelligence in the diagnosis of PCOS is highly promising in terms of conducting personalized therapies along with detecting PCOS at an early stage. Additional studies are necessary to comprehend the pathogenesis in order to provide a precise diagnosis.

## Figures and Tables

**Table 1:** The four PCOS phenotypes are based on the Rotterdam criteria (2003). “*” refers to the presence of the symptoms.

	Anovulation/Oligo-Ovulation	Hyperandrogenism	PCOM
Phenotype A	*	*	*
Phenotype B	*	*	
Phenotype C		*	*
Phenotype D	*		*

**Table 2:** Evolution of elements of diagnostic criteria for PCOS.

	NIH 1999	ROTTERDAM 2003	AE-PCOS SOCIETY 2006
	Both elements are needed:	2 of 3 elements are needed:	Both elements are needed:
1	Chronic anovulation	Oligo- and or anovulation	Oligo-anovulation and/or polycystic ovarian morphology
2	Clinical and/or biochemical signs of hyperandrogenism	Clinical and/or biochemical signs of hyperandrogenism	Clinical and/or biochemical signs of hyperandrogenism
3	-	Polycystic ovarian morphology	-
